# Estimation of Access to Safe Drinking Water to Households in Etawah District: A Cross-Sectional Study

**DOI:** 10.7759/cureus.47154

**Published:** 2023-10-16

**Authors:** Deepanshi Saxena, Prashant K Bajpai, Dhiraj K Srivastava, Sushil K Shukla, Pankaj K Jain, Nilima Takhelchangbam

**Affiliations:** 1 Community Medicine, Sarojini Naidu Medical College, Agra, IND; 2 Community Medicine, Uttar Pradesh University of Medical Sciences, Etawah, IND; 3 Medicine, King George's Medical University, Lucknow, IND; 4 Community Medicine, King George's Medical University, Lucknow, IND; 5 Community Medicine, Maharani Laxmi Bai Medical College, Jhansi, IND

**Keywords:** water supply/standards, environmental health, qualitative, wash, drinking water quality

## Abstract

Context: Unsafe drinking water causes diarrheal disease and environmental enteropathy. The quality of water is determined by its physical, chemical, and biological characteristics. Water sources have a significant impact on household members' health, particularly children. To combat this, India is committed to providing household tap connections to ensure the delivery of safe drinking water with the "Jal Jeevan Mission."

Aims: This study aims to estimate the access to safe drinking water and the physical and chemical qualities of water (qualitatively) in the urban and rural areas of Etawah district, India.

Settings and design: A cross-sectional study was conducted in Etawah district from January 2020 to December 2021. The study subjects were the eldest female of the family. A total of 312 females were included. The data collected were analyzed using IBM SPSS Statistics for Windows, version 25 (released 2017; IBM Corp., Armonk, New York, United States) for descriptive analysis.

Results: In the present study, 76.3% (238/312) of households in the urban and rural areas had access to safe drinking water (here, the meaning of the word "safe" is based on its operational definition). A total of 130 (83.3%) households in rural areas and only 21 (13.5%) in urban areas had private supply as the primary water source. The physical and chemical qualities of water were within the requirement (acceptable limit) and permissible limit in all the study areas, so the water is considered safe for drinking.

Conclusions: This study reported that 76.3% (238) households had access to safe drinking water according to the operational definition. The major public source of drinking water was public-supplied tap water, and in private sources, submersible or boreholes were the most common.

## Introduction

According to the Joint Monitoring Programme 2021 Annual Report, one in four people lacked safely managed drinking water services, 367 million used unimproved water sources, and 122 million drank surface water [1]. Goal 6 of the 2030 Agenda for Sustainable Development aims for universal access to safely managed drinking water service (6.1.1). Access to safe water is a human right [2,3].

Unsafe drinking water and poor sanitation and hygiene cause diarrheal disease and environmental enteropathy, inhibiting nutrient absorption, resulting in undernutrition [4]. However, undernutrition is most dangerous in developing countries while coping with other disease burden [5]. A study revealed that both piped water and improved water overall increased between 2000 and 2017 [6].

Despite the World Health Organization's drinking water quality guidelines [5], for safe drinking water, water contamination from diverse sources has increased in most countries in recent decades. In this purview, India launched the Jal Jeevan Mission (August 2019) committed to providing "Har Ghar Jal" by 2025 to all households in rural India [7,8]. Hence, considering the hypothesis that there is no safe drinking water supply, this study was conceived to estimate the access to safe drinking water and the physical and chemical qualities of water (qualitatively). The study also estimated the proportion of primary and secondary sources of safe drinking water.

## Materials and methods

This study is a part of a larger study and is a community-based cross-sectional study with the study period being January 2020 to December 2021, conducted in the rural and urban field practice areas of the Department of Community Medicine, Uttar Pradesh University of Medical Sciences, Saifai, Etawah district, Uttar Pradesh, India. The field practice areas were selected purposively because of the feasibility of reaching there.

Sample size

According to the fourth National Health Family Survey (NFHS-4), the percentage of households with improved drinking water sources was 96.4% [9]. Therefore, considering the 3% absolute precision at a 95% confidence interval (CI) and applying them and adding 5% of 148 for loss to follow-up (here, 5% was taken instead of 10% because of financial constraints), the total sample was 156. The sample size was rounded off. Therefore, a sample size of 156 was taken each from the urban and rural areas to ease the comparison. Accordingly, a total of 312 sample sizes were taken.

**Figure 1:**
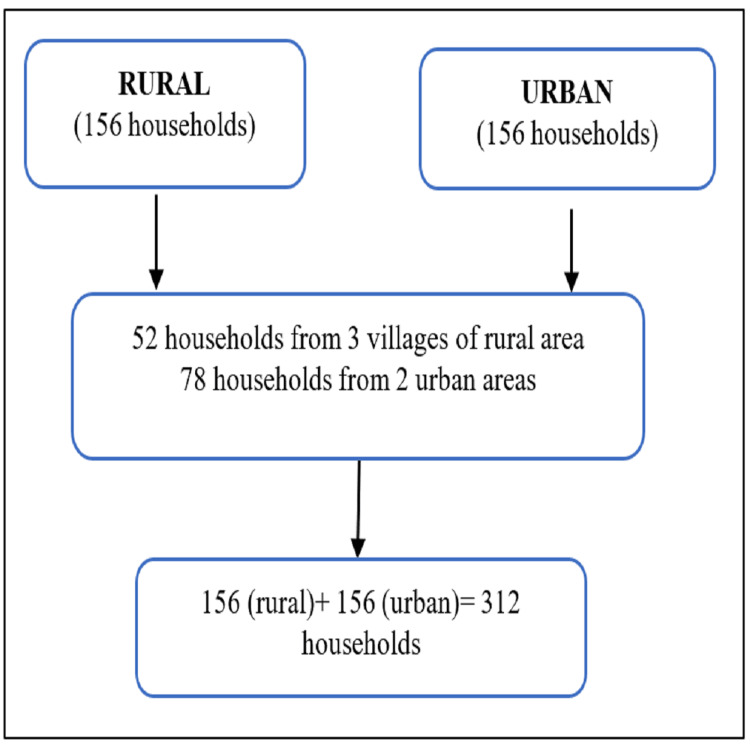
Flowchart of the methodology of data collection

The rural field practice area includes three villages (Geenja, Ujhiyaini, and Baghuiya), and the urban area includes two colonies (Kashiram colony and Madhaiya Shiv Narayan). The current updated list of households was obtained from the Department of Community Medicine. For selecting the households, a systematic random sampling method was applied until the target sample size was achieved, and the first household was selected by the currency note method for all the areas (Figure 1). From each of the three villages, 52 households were selected for the survey to achieve a total sample of 156 households. Similarly, from each of the two urban areas, 78 households were selected.

Ethics approval of research

Ethical clearance was duly taken from the Institutional Ethical Committee (approval no. ID-84/2019-20) before proceeding with the study.

Study participants and eligibility criteria

The study participants were the eldest female of the family, actively involved in kitchen chores, and residents of the field practice areas. They were included as they are most responsible for these works in the country. In the absence of female members, widowers or divorced males or male partners living far from their female partners were included. If the respondents were not available, then a revisit was arranged later. Those who did not give their consent or were not available after the revisit were excluded. There were no missing data or exclusions in this study.

Data collection tool

All information was gathered by interviewing the study participants using a predesigned, pretested, and semi-structured questionnaire with three sections. The first was for the sociodemographic profile, which included the name, age, religion, caste, type of family, number of children, number of family members, socioeconomic status of the family, education, income, marital status, and occupation of the study subjects. The second section comprises questions related to drinking water sources, namely, accessibility, availability (in terms of time), continuity of water from primary and secondary sources, time required to fetch water, reasons for nonavailability, and drainage system.

To determine the proportion of households having access to safe drinking water, the respondents were inquired whether the primary source of drinking water is from a private supply, government piped water, government tap water, hand pumps, or any other source. The secondary source (any source, i.e., submersibles, government taps, or neighbor's house) was considered when a household takes drinking water when the water is not available in the primary source (Table 1).

**Table 1:** Variables and outcome measures

Variables	Outcome measures
Sources of safe drinking water	Primary source/secondary source
Primary source	Private supply, government piped supply, government taps, hand pumps, or any other source (from the neighbor)
Availability of water	<12 hours and >12 hours
Access to safe drinking water	The proportion of households having access
Water physical and chemical quality (qualitative) (Environmental Protection Agency (EPA) standards)	Required (acceptable limit) and permissible limit in the absence of an alternate source

**Figure 2:**
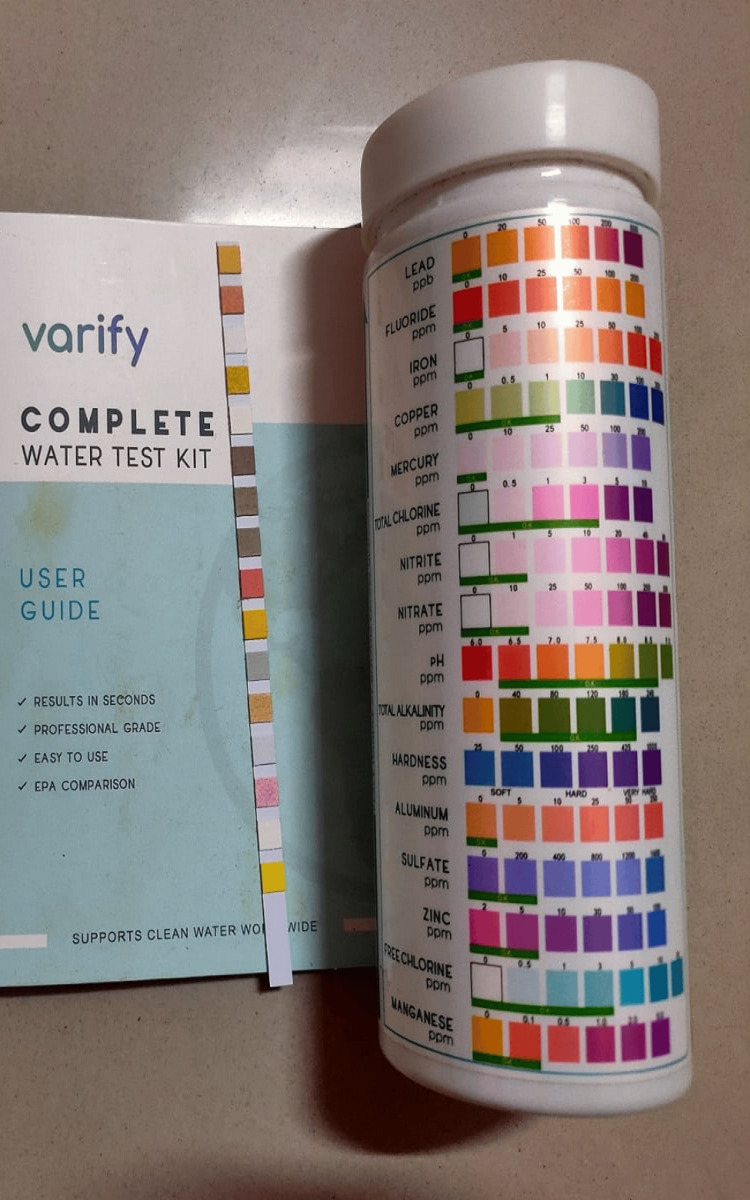
Image of the water testing strip kit (Varify)

The third section was for the questionnaire on water quality assessment, and the outcome was measured by the water's physical and chemical qualities. The physical qualities analyzed include color, turbidity, odor, and total dissolved solids, and the chemical qualities analyzed were lead, fluoride, iron, copper, mercury, total chlorine, nitrite, nitrate, pH, total alkalinity, hardness of water, sulfate, zinc, and manganese. The water testing strip kit (Varify, USA), based on Environmental Protection Agency (EPA) standards, which were already color-coded, were used (Figure 2).

**Figure 3:**
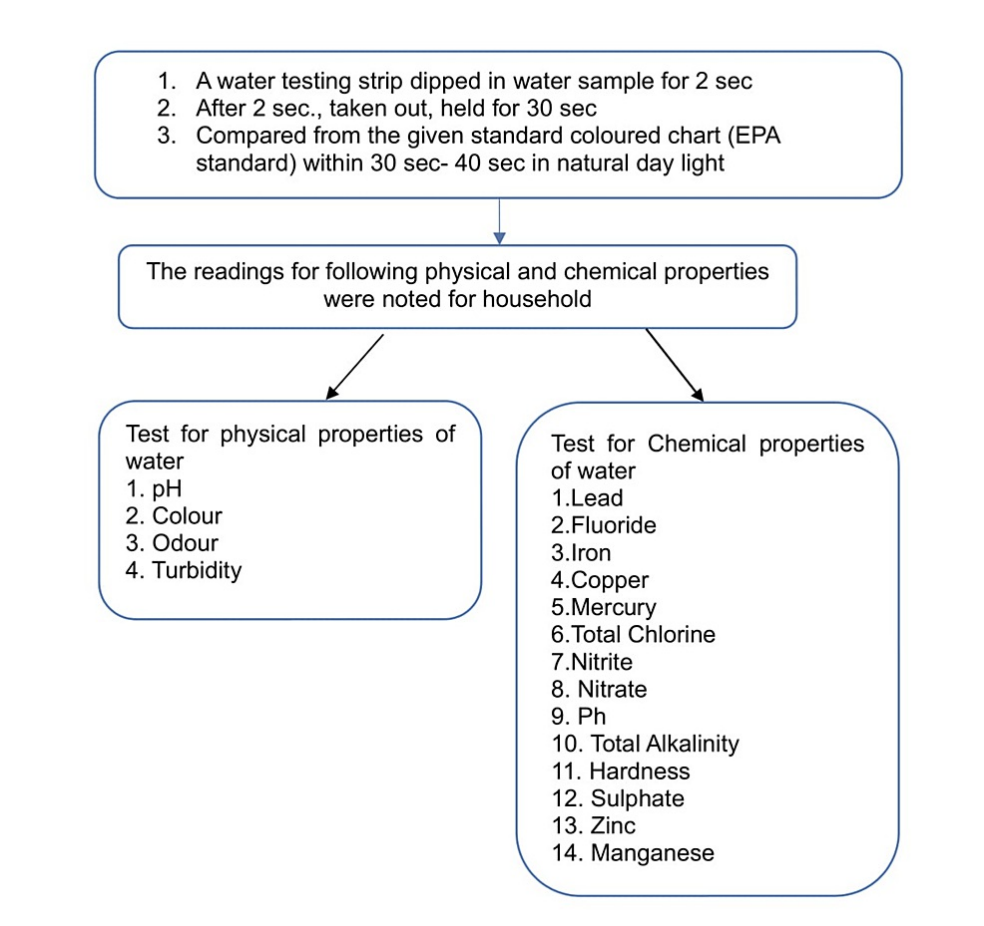
Flowchart for the methodology of water sample testing

The strip was dipped for two seconds in the water sample, which was taken directly into any household's utensil, from the primary source where the household filled the water. After 30 seconds, the strip was taken out. The colors on the strip changed according to the parameter value. The strip was matched to the standard color chart in the daylight within 30-40 seconds. The reading against them was noted and analyzed to find out if the drinking water has an acceptable or permissible limit (in the absence of an alternate source) (Figure 3).

Pilot testing of the questionnaire

The questionnaire was translated into the local dialect, i.e., Hindi, and forward-translation and back-translation methods were implemented. For internal consistency, the reliability test using Cronbach’s alpha (0.736) was done on the data of the pilot study. If any information was missing any particulars, the respective study subject was contacted again on the next day. There may be confounders or effect modifiers, but the study had not considered them.

Operational definition

Safe or safely managed drinking water is defined as drinking water from an improved water source that is located on premises, available when needed, and free from prior chemical and microbiological contaminations. Improved sources include piped water, boreholes or submersibles, public taps or standpipes, and packaged water. Government piped water supply is a "household connection," a piped water supply connected with in-house plumbing to one or more taps (e.g., in a kitchen or bathroom) [7,10]. Here, a source refers to the point from which water is collected (e.g., taps or boreholes/hand pumps) and not the origin of the water supplied (e.g., surface water or groundwater).

Here, for operational definition, safe drinking water is water from an improved water source that is located on premises, available for more than 12 hours, and free from prior chemical contaminations or within the required (acceptable limit) or permissible limit in the absence of an alternate source according to the EPA standards. The microbiological contamination could not be determined due to overburdened laboratories by COVID-19 samples, which is the limitation of this study.

Data collection

Data collection was done, taking all the COVID-19 appropriate behavior and analysis into consideration, from May 2021 to November 2021. Written and informed consent was taken, and confidentiality and privacy were ensured at all stages during the study. The data collected were entered into a Microsoft Excel sheet and analyzed using IBM SPSS Statistics for Windows, version 25 (released 2017; IBM Corp., Armonk, New York, United States) for descriptive analysis.

## Results

In the present study, 156 households from each urban and rural area were involved, garnering a total of 312 participants. The sociodemographic characteristics of the study participants (N=312) for the urban and rural areas show that there were 52 participants each in the rural areas, i.e., Geenja, Ujhiyaini, and Baghuiya. Meanwhile, the total number of participants in the urban areas was 156, i.e., 78 each in Kashiram colony and Madhaiya Shivnarayan. The majority of the participants in the rural areas (69, 44.2%) and urban area (51, 32.7%) are 26 to 35 years old (Table 2).

**Table 2:** Sociodemographic characteristics of the study participants (N=312) Others*: unskilled, Others#: widow, **Modified BG Prasad scale (All India Consumer Price Index (AICPI) up to April 2020)

Characteristics		Rural (n=156) Frequency (%)	Urban (n=156) Frequency (%)
Age group (In years)	19-25	21(13.5)	15(9.6)
	26-35	69(44.2)	51(32.7)
	36-45	42(26.9)	50(32.1)
	46-55	13(8.3)	26(16.7)
	56 and above	11(7.1)	14(9.0)
Religion	Hindu	156(100.0)	138(88.5)
	Muslim	0(0.0)	18(11.5)
Type of family	Nuclear	69(44.2)	106(67.9)
	Joint	51(32.7)	33(21.2)
	Three generation family	36(23.1)	17(10.9)
Occupation	Housewife	148(94.9)	151(96.8)
	Others^*^	8(5.1)	5(3.2)
Marital status	Unmarried	18(11.5)	2(1.3)
	Married	132(84.6)	143(91.7)
	Others^#^	6(3.8)	11(7.1)
Educational status	Illiterate	42(26.9)	26(16.7)
	Primary school	23(14.7)	42(26.9)
	Secondary school	80(51.3)	87(55.8)
	Graduate	11(7.1)	1(0.6)
Socioeconomic status^**^	Upper class	44(28.2)	57(36.5)
	Upper middle	52(33.3)	36(23.1)
	Lower middle	40(25.6)	29(18.6)
	Upper lower	15(9.6)	23(14.7)
	Lower	5(3.2)	11(7.1)

A total of 130 (83.3%) households from the rural areas and only 21 (13.5%) from the urban areas had private supply as the primary water source (Table 3).

**Table 3:** Distribution of the drinking water from the primary source (N=312) Others*: from neighbor and public source; packaged drinking bottles

Primary source	Rural (n=156) Frequency (%)	Urban (n=156) Frequency (%)
Private supply	130(83.3)	21(13.5)
Public supply: supply in household	0(0.0)	135(86.5)
Others^*^	26(16.7)	0(0.0)

**Figure 4:**
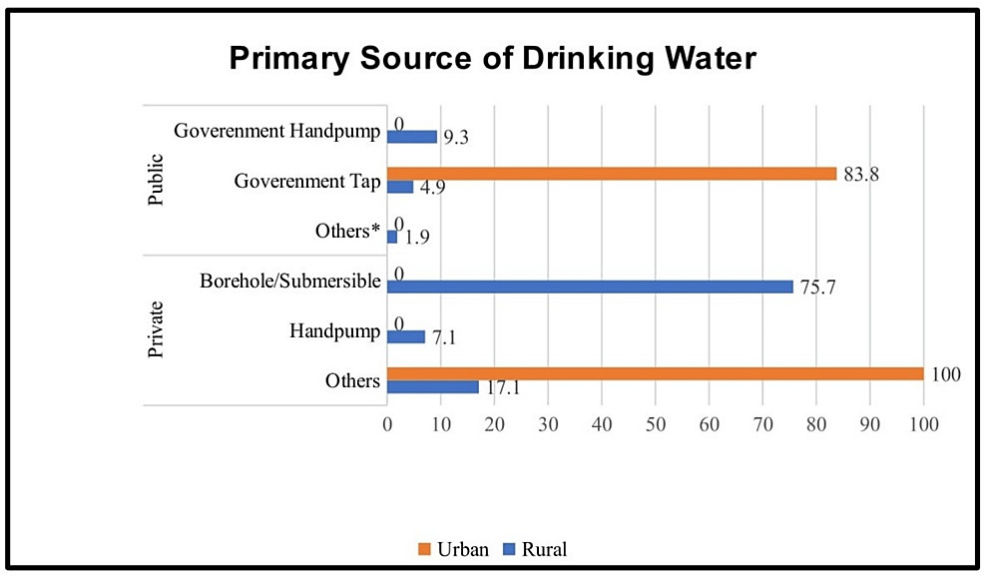
Distribution of types of drinking water as the primary source (N=312) Others*: from neighbors; Others: from neighbors, government taps and hand pumps, and packaged water bottles

Among the households, the primary water source in private supply was boreholes/submersibles (106, 75.7%), which was the most common type of water source in the rural areas. However, in the urban areas, most people (21, 100%) were arranging drinking water on their own (neighbors, government taps and hand pumps, and packaged water). Among the households that used public supply as the primary water source, most households (138, 83.8%) were using government taps (Figure 4).

In the rural areas, drinking water was unavailable in the primary sources for greater than 12 hours, and the reason for the unavailability was electricity problems in 47 of 156 (30.1%) households, followed by strict timings, i.e., punctual timings but for less duration, in three of 156 (1.9%) households. However, drinking water was unavailable from the primary sources for greater than 12 hours in urban areas, and the most common reason was strict timings (24, 15.4%). In the rural and urban areas, the secondary sources of drinking water were mostly from their own hand pumps (37, 23.7% and 12, 7.7%, respectively) when water was not available in the primary sources.

The physical quality of drinking water in the urban areas was found to be in the pH range of 7-7.5 in 104 (66.6%) samples of water. The physical quality of water was within the requirement (acceptable limit) in all the study areas and thus considered safe for drinking (Table 4).

**Table 4:** Physical quality of drinking water samples of the study area (N=312) *Requirement (acceptable limit)

Physical quality variables	Categories	Rural (n=156) Frequency (%)	Urban (n=156) Frequency (%)
Color	Clear^*^	156(100)	156(100)
Turbidity	No^*^	156(100)	156(100)
Odor	No^*^	156(100)	156(100)
^pH*^	6.5-7	152(97.4)	0(0)
	7-7.5	4(2.6)	104(66.6)
	7.5-8	0(0)	52(66.7)

In all the samples (312, 100.0%), lead, fluoride, iron, copper, mercury, total chlorine, nitrite, zinc, and manganese levels were within the requirement (acceptable limit). The permissible limit (250-425 ppm) of the sulfate level was found in 152 (97.4%) samples of the rural areas. Thus, the chemical quality of water was within the requirement (acceptable limit) and permissible limit in all the study areas and hence considered safe for drinking (Table 5).

**Table 5:** Chemical quality of drinking water samples of the study area (N=312) *Requirement (acceptable limit); #Permissible limit in the absence of an alternate source

Chemical quality of drinking water	Values	Rural (n=156) Frequency (%)	Urban (n=156) Frequency (%)
Lead	0-5 ppb^*^	156(100.0)	156(100.0)
Fluoride	0-4 ppm^*^	156(100.0)	156(100.0)
Iron	0-0.3 ppm^*^	156(100.0)	156(100.0)
Copper	0-0.1 ppm^*^	156(100.0)	156(100.0)
Mercury	0-0.002 ppm^*^	156(100.0)	156(100.0)
Total chlorine	0-0.5 ppm^*^	156(100.0)	156(100.0)
Nitrite	0-1 ppm^*^	156(100.0)	156(100.0)
Nitrate	0-10 ppm^*^	72(46.2)	156(100.0)
	10-25 ppm^*^	84(53.8)	0(0)
Total alkalinity	120-180 ppm^*^	4(2.6)	104(66.7)
	180-240 ppm^#^	152(97.4)	52(33.3)
Hardness	100-250 ppm^*^	4(2.6)	156(100.0)
	250-425 ppm^#^	152(97.4)	0(0)
Sulfate	0-200 ppm^*^	4(2.6)	156(100.0)
	200-400 ppm^#^	152(97.4)	0(0)
Zinc	0-5 ppm^*^	156(100.0)	156(100.0)
Manganese	0-0.05 ppm^*^	156(100.0)	156(100.0)

As the households are taking water from safe or safely managed drinking water sources as primary and secondary sources, 76.3% (238/312) of households in the urban and rural areas had access to safe drinking water according to the operational definition.

## Discussion

Safe drinking water is one of the most important public health concerns. To achieve the Sustainable Development Goals, access to safe water, in sufficient quantity and quality, is a prerequisite.

The present study revealed that the proportion of households having access to safe drinking water in rural and urban areas of the field practice areas was cent percent. However, in the rural areas, safe drinking water was being accessed privately, arranged by themselves. This was because of the incomplete construction of pipelines from the water tank to the households. The earlier NFHS-4 [9] reported that 96.4% households in Uttar Pradesh had access to safe drinking water; however, the recent report of NFHS-5 (2019-2021) revealed that the percentage is 99.2% [11].

In a study conducted by Joshi et al. in the urban slums of New Delhi in 2014, Kuberan et al. in Thandalam village, Chennai, and Pachori et al. in Tamil Nadu, the proportion of accessibility to safe drinking water was 100%, 99.0%, and 85.3%, respectively [12-14]. In the same way, in a study conducted by Bharti et al. in the slum households of Siliguri Municipal Corporation and by Kong et al. in the urban and rural localities of Malaysia, a developing country like India, the proportion of accessibility to safe drinking water was 92.1% and 96.2%, respectively [15-16]. Hence, the results of the various studies showed the strong political commitment and determined implementation of the program by the government. The present study demonstrated that the most common primary source of drinking water was piped water supply in urban areas. The results of this study were also consistent with the findings of Ssemugabo et al., i.e., 76.7% (303/395). The present study revealed that the most common primary source of drinking water in rural areas was boreholes or submersibles. The findings were almost similar to the study by Tussupova et al., i.e., 51.9% [17-18]. The similarity in results may be due to the active working government in the area and high political commitment.

The most common secondary source of water supply in the rural and urban areas in this study was private hand pumps (49, 15.7%). The drinking water in the primary source was not available for less than 12 hours in the field practice area (106, 33.9%). The reasons found were electricity problem, strict timings, and out-of-order water supply source. In a similar study by Kuberan et al. [12], 2% of households reported unavailability of drinking water for less than 24 hours. The reason mentioned by them was almost similar to the present study, i.e., strict supply timings, irregular supply, and unclean and bad water.

This study assessed the quality of drinking water. In the present study, the physical quality of drinking water (color, turbidity, odor, and pH) was within the acceptable limit in all the water samples. This result indicated that the water was suitable for drinking. 

A pH value of 6.5-7 was obtained in the majority of the water samples in the rural areas (152, 97.4%). A pH value of 7-7.5 was obtained in 108 (34.6%) samples. This finding is almost the same as the study by Shrivastava et al. in Varanasi and by Bedi et al. in Ludhiana, where the pH value was 7.3-7.7 [19,20]. Similar results were also obtained in a study conducted by Swaroop et al. in the Unnao district of Uttar Pradesh, where the pH value was within the acceptable limit [21]. In this study, the public water supply in the primary source was 51.6% (161) in the field practice areas. The findings were similar to those of Joshi et al., i.e., 11 (53%) [21].

In this study, the values for total alkalinity were found within permissible limits in almost all areas. In addition, the hardness and sulfate were within the permissible limits in all the rural areas. This may be due to the geographical location and non-treated water. The total alkalinity levels were within the permissible limits in a study by Shrivastava et al. in the Varanasi area. The hardness value was toward a higher range (227-869), which was in contrast with the present study. The sulfate levels were almost similar to the present study [19]. In the study by Swaroop et al., the alkalinity concentration ranged from 200 to 500, which was at a higher range and in contrast with the results of this study. This may be due to the contamination of water. The hardness level was more than the permissible limit (167-421), which was also in contrast with the results of this study [21].

The study by Bedi et al. showed that the alkalinity levels were slightly more than the permissible limit, while in the present study, it was within the acceptable limits, which was in contrast with this study. This may be due to the difference in the geographical area and the absence of treatment of drinking water [20].

In the present study, nitrite, nitrate, total chlorine, lead, fluoride, iron, copper, mercury, zinc, and manganese values in all the water samples were within the acceptable limits. Bedi et al., Shrivastava et al., and Swaroop et al. showed similar findings for the nitrate levels. However, the iron levels were found on the higher side. This may be due to the spatial location of the study area [19-21].

Limitations and way forward

The results and generalizability of this study should be interpreted considering the study’s limitations. The study areas were selected purposively as there were time and financial constraints. There may be subjective bias in testing the water samples by test strips as the results have to be interpreted based on the color change in the test strips. This study paves a way forward toward assessing the microbiological water quality of the drinking water as it could not be done due to COVID-19 restrictions. A further study is recommended to find out the effects on health by the quality of drinking water and drinking water practices in the field practice areas. The bacteriological test of drinking water will aid in this. Hopefully, all these areas where the people are not able to get government piped safe water supply will be covered by its target year.

## Conclusions

This study will act as a baseline and aid information regarding access to safe drinking water in the current data of NFHS-5. This research was specifically conducted in the Etawah district of India, providing region-specific insights, and is thus valuable for tailoring interventions and policies to address the unique challenges faced by different communities, contributing to public health efforts at the grassroots level. The results showed that the study achieved its objectives. This study reported that all households had access to safe drinking water according to the operational definition. However, the major supply of drinking water was either from public-supplied tap water that is the pipeline water for private submersibles or boreholes.
